# Enablers of Post-Validation Surveillance for Lymphatic Filariasis in the Pacific Islands: A Nominal Group Technique and Expert Elicitation

**DOI:** 10.3390/tropicalmed11020062

**Published:** 2026-02-23

**Authors:** Adam T. Craig, Clement Couteaux, Ken Jetton, Roger Nehemia, Oliver Sokana, Tanebu Tong, Temea Bauro, Taulanga Baratio, Ofa Tukai, Joe Takai, Satupaitea Viali, Noel Gama Soares, Maria Ome-Kaius, Mary Yohogu, Litiana Volavola, Patricia Tatui, Fasihah Taleo, Salanieta Saketa, Andie Tucker, Charles Mackenzie, Katherine Gass, Holly Jian, Colleen L. Lau, Harriet L. S. Lawford

**Affiliations:** 1 Operational Research and Decision Support for Infectious Diseases (ODeSI) Program, Centre for Clinical Research, The University of Queensland, Royal Brisbane Hospital, Herston, QLD 4029, Australiacolleen.lau@uq.edu.au (C.L.L.);; 2 Agence de Santé of Wallis and Futuna, Sia Hospital, Hahake District, Wallis 98600, Wallis and Futuna; 3 Republic of the Marshall Islands Ministry of Health and Human Services, P.O. Box 16, Majuro 96960, Marshall Islands; 4 Te Marae Ora Cook Islands Ministry of Health, Avarua P.O. Box 109, Rarotonga, Cook Islands; 5 Solomon Islands Ministry of Health and Medical Services, Honiara P.O. Box 349, Solomon Islands; 6 Kiribati Ministry of Health and Medical Services, Tarawa P.O. Box 268, Kiribati; 7 Tonga Ministry of Health, Nuku’alofa P.O. Box 59, Tonga; 8 Oceania University of Medicine, Apia P.O. Box 232, Samoa; 9 School of Medicine, Faculty of Health Sciences, National University of Samoa, Toomatagi, Apia P.O. Box 1622, Samoa; 10 Timor-Leste Ministry of Health, Palaco do Governo, Dili TL10001, Timor-Leste; 11 Papua New Guinea Institute of Medical Research, Goroka P.O. Box 60, Papua New Guinea; 12 Papua New Guinea Ministry of Health, Waigani 121, Port Moresby P.O. Box 807, Papua New Guinea; 13 Fiji Ministry of Health and Medical Services, Dinem House, 88 Amy Street, Toorak, Suva P.O. Box 2223, Fiji; 14 Niue Department of Health, Niue Foou Hospital, Alofi P.O. Box 40, Niue; 15 World Health Organization Vanuatu Country Office, Port Vila P.O. Box 177, Vanuatu; 16 Public Health Division, Pacific Community, Suva P.O. Box 2223, Fiji; 17 Global Alliance to Eliminate Lymphatic Filariasis, Decatur, GA 30030, USA; 18 Task Force for Global Health, Decatur, GA 30030, USA

**Keywords:** lymphatic filariasis, neglected tropical diseases, post-validation surveillance, Pacific Islands, elimination, nominal group technique, health systems

## Abstract

Lymphatic filariasis (LF) is a mosquito-borne neglected tropical disease that causes substantial morbidity and social exclusion. Global efforts under the World Health Organization’s Global Programme to Eliminate Lymphatic Filariasis have markedly reduced prevalence, and several Pacific Island Countries and Territories (PICTs) have achieved elimination of the disease as a public health problem. However, post-validation surveillance (PVS), essential for detecting resurgence and enabling early response, has rarely been implemented, and barriers to its delivery remain poorly understood. We used two complementary qualitative approaches to identify systemic barriers and enablers to LF PVS in PICTs. First, we conducted a Nominal Group Technique followed by a structured expert elicitation involving program managers and technical staff. Data were analysed thematically and triangulated across sources. Participants identified 70 challenges which were consolidated into ten thematic domains. Pertinent barriers relate to limited leadership understanding of LF and surveillance options, inconsistent technical and financial support, and a lack of context-appropriate operational guidance. Additional challenges included limited field-ready diagnostics, procurement delays, the absence of formal mandates, and low community engagement. Enablers included embedding PVS within existing health services, leveraging trusted community networks, strengthening regional frameworks, and co-developing practical tools with countries. Sustaining LF elimination in the Pacific will require political commitment, regional collaboration, and integrated, programmatic approaches informed by recent PVS experience.

## 1. Introduction

Lymphatic filariasis (LF) is a mosquito-borne neglected tropical disease (NTD) caused by three species of filarial worms: *Wuchereria bancrofti*, *Brugia malayi*, and *B. timori*, and transmitted to humans by mosquito vectors, including Aedes, Culex, and Anopheles species [1]. Globally, *W. bancrofti* accounts for about 90% of infections [2]. Adult worms live in the lymphatic system, where untreated infections can lead to progressive and irreversible lymphatic damage. The clinical consequences, such as severe lymphoedema, skin thickening and hydrocele, are associated with pain, disability, and significant social and economic exclusion [3,4].

In 2018, an estimated 51 million people were living with LF, mainly in Africa and South Asia. This indicates a 74% reduction in prevalence since the World Health Organization’s (WHO) Global Programme to Eliminate Lymphatic Filariasis (GPELF) was launched in 2000 [5]. The decline is widely attributed to sustained national efforts to interrupt transmission and reduce disease burden through mass drug administration (MDA) and morbidity management [6]. Nevertheless, despite this notable progress, the remaining burden and the risk of resurgence in settings that have eliminated LF call for renewed focus on surveillance and long-term programme sustainability [7,8].

**Figure 1:**
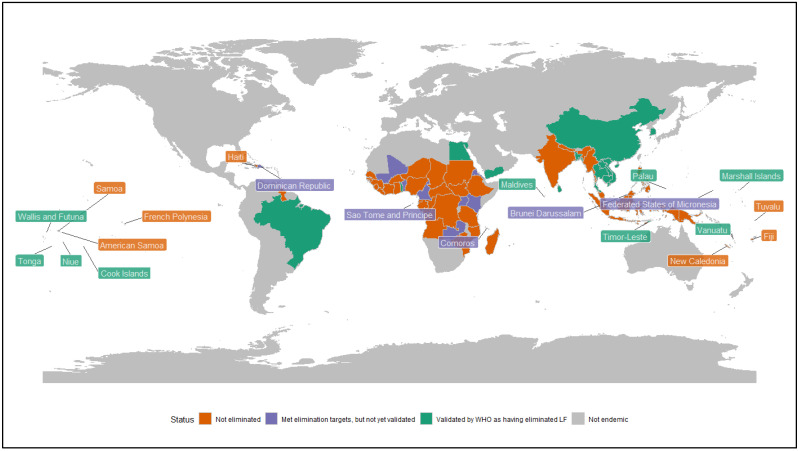
Lymphatic filariasis endemic countries and territories, and their elimination status as of 2026.

WHO has established a formal validation process for countries that meet LF elimination thresholds. The 2011 guideline criteria (which were updated in 2025, but are yet to be applied) defined the LF elimination threshold as <2% Ag prevalence in areas in which *W. bancrofti* is endemic and *Anopheles* or *Culex* is the vector, <1% Ag prevalence in regions in which *W. bancrofti* is endemic and *Aedes* is the primary vector, and <2% antibody (Ab) prevalence in areas were *Brugia* spp. are endemic [9,10]. To improve sensitivity, the 2025 LF surveillance guidelines [10] suggest that <1% *Ag* (*W. bancrofti*) and *Ab* (*Brugia* spp.) prevalence target threshold for all vector and parasite species. Countries seeking validation of having eliminated the disease as a public health problem submit a dossier of evidence to WHO, including results of epidemiological surveys, plans for morbidity management, and a commitment to implement post-validation surveillance (PVS). Post-validation surveillance refers to the systematic collection, analysis, and use of epidemiological, entomological, and programmatic data undertaken after a country has been validated as having eliminated LF as a public health problem, with the primary objective of detecting any resurgence of transmission early and enabling a timely public health response [10]. As of September 2025, 21 (of 72) LF-endemic countries have received WHO validation for elimination as a public health problem, with a further 14 having completed mass drug administration (MDA) and are under surveillance but have not yet been validated. 38 other LF endemic countries continue to implement MDA to reduce the prevalence of the disease, and one has yet to start MDA [11] (Figure 1).

Sixteen of the 22 Pacific Island Countries and Territories (PICTs) have reported LF transmission. Among these, eight (Cook Islands, Kiribati, Niue, the Republic of the Marshall Islands, Palau, Tonga, Vanuatu, and Wallis and Futuna) have gained WHO validation for eliminating LF as a public health problem. Although each of these countries submitted PVS plans as part of the validation process, plan implementation remains limited. The reasons behind inaction are not well understood; without urgent efforts to close this knowledge gap, advancing PVS will be challenging risking decades of elimination work.

In 2025, the WHO issued new guidelines for the design and implementation of LF surveillance [10]. These guidelines mark a significant shift towards clearer global expectations for the surveillance activities that countries working towards sustained LF elimination should implement. This paper directly responds to this development. It aims to identify the core barriers to PVS implementation in the PICT context and to gather implementer-led solutions that can drive policy and operational changes. The insights here are grounded in the perspectives of national program managers and field staff. They are relevant not only to the Pacific but also to countries currently implementing or preparing to implement PVS. Building on our earlier conceptual work [12], this study offers practice-oriented evidence to inform the next phase of global LF elimination efforts.

## 2. Materials and Methods

### 2.1. Ethical Consideration

Ethical approval for this study was obtained from The University of Queensland Human Health Ethics Review Committee (Project #2024/HE000224). Written informed consent was secured from participants prior to their participation.

### 2.2. Study Design and Research Questions

This study used two complementary qualitative methods to examine the views of national program managers and frontline staff on the systemic barriers and enablers to implement PVS for LF in PICTs. The methods included a Nominal Group Technique (NGT)-based consultation in 2024, and an expert workshop held in 2025.

### 2.3. Nominal Group Technique

A range of consensus-based and participatory methods exist to elicit stakeholder priorities, including surveys, focus groups, citizen juries, discrete choice experiments, and Delphi techniques [13,14]. Among these, consensus group methods are especially valuable when empirical evidence is limited or contested. Widely used across sectors such as education, engineering, management, and health, these methods have informed the development of guidelines, diagnostic criteria, and program planning [15,16]. The NGT, developed by Delbecq and Van de Ven in 1971 [17], is a structured approach designed to facilitate idea generation, discussion, and prioritisation. NGT enables comparison of priorities across individuals and groups, highlighting areas of agreement and divergence. By design, the NGT promotes equal participation and minimises the influence of dominant individuals, thereby reducing measurement bias [16,18].

The NGT followed seven sequential steps, described in detail in a companion methods paper [19], and summarised here: (i) presentation of the nominal question; (ii) silent generation of individual ideas; (iii) round-robin generation of ideas; (iv) group discussion to clarify and elaborate; (v) individual scoring and rank of ideas; (vi) aggregation and presentation of collective rankings; (vii) open discussion to reflect on the results.

The NGT was held during the 2024 ‘Coalition for Operational Research on Neglected Tropical Diseases (COR-NTD) Pacific Islands’ meeting held in Brisbane, Australia in 25–26 September 2024. The nominal question, developed by two authors (AC and HL) following guidance from Mullen et al. [20], was, ‘What do you consider to be the most significant challenges you face, or anticipate facing, in implementing PVS for LF in your country or territory?’ For the scoring step (step 5), participants were allocated ten votes to distribute across identified challenges as they saw fit. Votes could be concentrated on a single issue or spread across multiple priorities. All notes and individual scoring sheets were collected and transcribed. Facilitators kept detailed records of the discussion, capturing participants’ views, insights, and supporting arguments.

### 2.4. Expert Workshop

A follow-up expert workshop was held during the ‘Voices and Visions: Building Partnerships for Integrated Surveillance of Lymphatic Filariasis and Other Infectious Diseases in the Pacific Islands’ meeting in Brisbane, Australia, from 8–10 July 2025. The workshop gathered 50 participants, including 15 from 14 PICTs and Timor-Leste. The workshop focused on advancing the implementation of PVS in the region. It featured themed discussions on aligning LF surveillance with PICTs’ context, operationalising LF PVS, and responding to PVS-generated surveillance signals. Sessions were audio-recorded and transcribed for later analysis.

### 2.5. Data Analysis

Quantitative ranking data generated during the NGT were summarised descriptively. Qualitative data from both the NGT and the expert workshop were analysed using a rigorous, iterative, inductive thematic approach [21]. Transcripts and facilitator notes from both activities were independently reviewed and coded by two authors (AC and HL) to enhance analytic credibility. Codes were compared, discussed, and systematically grouped to identify dominant and cross-cutting themes. Differences in interpretation were resolved through reflexive discussion; where consensus could not be reached, alternative interpretations were retained to preserve analytic transparency. Preliminary themes were then shared with participants for validation, feedback, and iterative refinement, strengthening the trustworthiness of the findings.

To translate the barriers identified into actionable guidance, we developed a structured framework informed by the Action–Actor–Context–Target approach [22]. This framework delineates specific suggested actions required to address each barrier, identifies the actors responsible for implementation, and proposes measurable indicators to monitor progress. In doing so, it provides a practical blueprint to help policymakers plan, resource, and evaluate PVS activities.

### 2.6. Reflexivity Statement

The authors, a consortium of academics, advocates, and national programme implementers, acknowledge that their prior conceptual and applied work on lymphatic filariasis post-validation surveillance in the Pacific Islands both motivated the conduct of this study and may have influenced the interpretation of its findings. To minimise the influence of pre-existing assumptions on data interpretation, several steps were taken. First, the NGT and expert workshop were structured to prioritise participants’ priorities and limit researcher direction. Second, qualitative data were independently coded with reflexive discussion used to interrogate assumptions and resolve differences in interpretation. Where consensus could not be reached, divergent views were retained during the early stages of iterative group analysis. Third, preliminary themes were shared with participants for validation and feedback, enabling corrections, clarifications, and refinements of interpretations. Together, these measures were intended to enhance analytic rigour, transparency, and trustworthiness while acknowledging the researchers’ positionality within the field.

## 3. Results

### 3.1. Participant Characteristics

Eight individuals (one female and seven males) from six PICTs participated in the NGT, and 12 individuals (seven females and five males) from nine PICTs and Timor-Leste took part in the expert workshop. Three people were involved in both the NGT and the expert workshop. Collectively, participants had expertise in medical practice, nursing, epidemiology, surveillance, environmental health, program management, and academia.

The NGT aimed to identify the most significant challenges to implementing LF post-validation surveillance (PVS) in Pacific Island settings. Participants identified 70 discrete obstacles, underscoring the breadth and complexity of barriers to PVS implementation across the region. These were subsequently synthesised into ten overarching themes; the themes and corresponding voting results are presented in Supplementary Material Table S1.

The NGT ranking exercise demonstrated strong consensus, with all participants placing the same three themes in the highest-priority tier. The most frequently cited challenges were limited understanding of, or commitment to, PVS among national-level decision-makers; irregular and often short-term technical and financial support for PVS activities; and the absence of clear guidance on how to select and apply epidemiologically and contextually appropriate PVS strategies.

These themes were reinforced during the expert workshop, where participants emphasised the need to raise awareness among senior health leaders of both the importance of PVS and the risk of disease resurgence in its absence. Workshop participants also expressed concern about the feasibility of sustained, nationally led PVS implementation without a funded, long-term regional strategy for LF control.

These themes, and other emergent barriers and enablers, are explored in detail below. Table 1 maps each barrier domain to operational components structured according to the Action–Actor–Context–Target framework, thereby enhancing clarity, accountability, and measurability.

### 3.2. National Leadership, Understanding and Commitment to PVS

Participants in the NGT were unanimous in identifying limited awareness and engagement among national leaders as the most pressing barrier to PVS implementation; this was reiterated during the expert workshop discussion. The importance of this issue was underscored by the fact that it received more than twice as many votes as the next-highest-ranked challenge during the NGT and was a recurring theme throughout the workshop. Several participants explained that while technical discussions around prevalence thresholds, transmission modelling, and cost-effectiveness are essential, they are unlikely to result in meaningful action unless senior officials, including ministers, permanent secretaries, and health directors, understand the ongoing risk of LF resurgence and the rationale for continued surveillance.

A common theme in the discussions was that the term “elimination” is often misunderstood by those not closely involved in LF work, leading to the belief that LF is no longer a threat. This misconception, often equating elimination with eradication, was cited as a key reason why PVS does not receive political or budgetary attention. In some contexts, the problem is compounded by translation challenges. Some participants noted that in their languages there is no clear distinction between the terms “eliminate” and “eradicate,” making effective communication and advocacy even more difficult.

Discussions during the NGT and subsequent expert workshop raised the possibility that international partners may need to adjust their support strategies. Rather than focusing on technical implementation or field activities, participants suggested the need for evidence that directly addresses policymakers’ information needs and presents an economic case for investing in LF PVS Participants suggested that to get ‘traction’ on LF PVS, it needs to be raised and endorsed by the regional health governance bodies, namely, at the Pacific Public Health Surveillance Network (PPHSN) and Pacific Heads of Health meetings. Given their secretariat functions, meeting participants felt that the WHO and the Pacific Community (SPC) are well-positioned to advocate for this.

### 3.3. Technical and Financial Support for PVS

Participants identified resource mobilisation as the second most critical barrier to effective PVS implementation. They described a noticeable shift from routine activities and funding under the Pacific Programme to Eliminate LF (PacELF) toward sporadic, externally driven, short-term projects. This change was interpreted as a decline in donor (and perhaps national leaders) interest in LF after countries achieve elimination targets.

Several participants emphasised that, without predictable financing, it was challenging to advocate and embed PVS activities within broader health system planning. Instead, it was said that programs were often left reliant on externally funded research projects that tended to be short-term and piecemeal in approach. As one participant explained, “Without reliable domestic or partner financing, PVS cannot be planned and hence stops.” Another noted that this situation risks turning PVS into a reactive or opportunistic activity rather than a routine, integrated part of public health systems.

Participants made clear that resourcing extends beyond money alone. It includes access to diagnostic kits, the availability of fieldwork equipment, including vehicles for transportation, and technical advice. What is most needed, they said, is predictability and stability to allow national health programs to organise and implement LF PVS.

In response to these concerns, participants proposed exploring ways to align PVS with established, well-funded and complementary programs, such as vaccination programs, and concepts, such as health systems strengthening and universal health coverage. Support from technical partners in developing cost-effectiveness arguments and investment cases was viewed as beneficial in encouraging both domestic and international donor financing.

### 3.4. Context-Specific Guidance and Support for PVS Implementation

Participants consistently emphasised that practical context-relevant guidance to support the implementation of PVS was critical. While global frameworks and recommendations exist, such as those from WHO, these were viewed as too general and not sufficiently aligned to the realities of small island settings. Specifically, participants noted gaps in advice related to designing and selecting surveillance strategies, how to advocate (domestically and internationally) for LF resourcing effectively, and determining triggers for public health action. In the absence of such guidance, participants expressed reluctance to advocate for PVS within their health systems. As one participant put it, “We don’t have anything to back up our requests… we don’t know if what we think is the best thing to do, and there is no one who can advise.” This uncertainty is reported to have contributed to program managers feeling under-supported in making technical decisions.

Participants at the expert workshop, where the new WHO guidelines for LF PVS [10] were introduced, acknowledged that the document addressed some previously identified needs. However, echoing the 2024 NGT findings, they emphasised the importance of guidance that reflects the practical realities of Pacific Island contexts. They recommended developing Pacific-specific tools, training materials, and case studies from other small island settings to translate global recommendations into more feasible strategies and to use these to advocate (domestically and with development partners and donors) for increased financial and technical support.

### 3.5. National Capacity and Workforce Development for PVS Implementation

Participants described a reliance on external agencies to fund and implement PVS, contrasting this with earlier successes under the Pacific Programme for the Elimination of Lymphatic Filariasis (PacELF) [23] where strong national leadership and sustained engagement contributed to the effective State-led implementation of LF elimination activities. The participants noted that the PacELF model of ownership should be extended to PVS; however, reduced funding, shifting leadership priorities, and competing demands across the health system have made it difficult to maintain domestic momentum, leading to greater reliance on external agencies.

To address these challenges, participants called for investment in national workforce development. Participants recommended that capacity-building be long-term and embedded within national systems, rather than delivered through one-off external workshops. Approaches such as mentoring, mirroring, and South-South exchange, supported by regional partners, were seen as helpful in building practical, applied expertise. Development partners were encouraged to continue their support, but in ways that support capacity building and the transition to country-led implementation.

### 3.6. Diagnostic Tools and Supplies

Participants raised concerns about the practicality of accessing diagnostic methods used in PVS, as well as the challenges of preparing and shipping blood slides for microscopic examination, should LF be detected. These procedures were viewed as logistically challenging to implement across many of the PICTs, where populations are dispersed across hundreds of islands, which lack supply chains, basic infrastructure (such as transport), and stock management, thereby inhibiting action. As one participant stated, “these issues are things that those from bigger countries don’t understand…they make delivery of public health service (including PVS) complicated to conduct.” To address this, participants suggested exploring alternative, more feasible diagnostic methods. STANDARD Q’s Filariasis Antigen Test (QFAT) and dry blood spot antibody testing were mentioned as options that may be easier to use and better suited to field conditions. Continued efforts to develop and validate field-ready diagnostic tools were considered essential research ventures to reduce the logistical burden of current testing methods and make PVS more feasible.

### 3.7. Integration and Community Engagement

Participants described a decline in community awareness and interest in LF following the completion of MDA campaigns and after receiving validation of elimination of the disease as a public health program. This reduced visibility was attributed to a gap in PVS public health communication and a shift in national health priorities toward more visible issues, such as non-communicable disease (NCD) prevention. Several participants of the expert workshop expressed concern that if new cases were identified during PVS, communities might misinterpret this as evidence that elimination efforts had failed and resist efforts to restart treatment.

In response, participants suggested that PVS should be reframed not as a standalone, disease-specific task but as part of a broader approach to health and wellbeing. Participants indicated that embedding PVS within routine services, such as maternal and child health programs, NCD screenings, and school-based health initiatives, which communities already value, could help maintain the acceptability of PVS efforts. This approach was seen to offer operational efficiencies by reducing duplication and aligning with activities that are better resourced and more regularly delivered.

Expert workshop participants pointed to recent examples of integrated programming, including the incorporation of LF surveillance within the WHO STEPwise approach to NCD risk factor surveys in Niue [24] and school- and health facility-based integration in Niue and Tonga [8,24,25]. They also suggest that integration with other seroprevalence surveys (e.g., those designed to monitor measles, rubella, and Hepatitis B elimination) be considered. Integration, as a model, was broadly seen as well-suited to small island contexts, where limited human resources demand efficient use of available systems. There was broad agreement that designing PVS activities with multiple benefits and visible community value was key to ensuring sustained engagement and long-term success.

### 3.8. Mandates and Accountability for PVS

Participants highlighted the lack of formal mandates or strict accountability measures as reasons for the deprioritisation of PVS within national health systems. It was noted that “without clearly defined requirements, LF PVS activities are seen as optional, and often overlooked in favour of other urgent health issues.” This observation echoes lessons from other disease elimination programs, where activities cease once formal monitoring is reduced.

To strengthen accountability, participants recommended that the need for PVS be more clearly articulated and that compliance monitoring and evaluation be integrated into routine health system indicator sets, with results reported in public health service delivery reports. Participants suggest that development partners, such as WHO, play an important advocacy role by encouraging and supporting countries to include PVS as part of health service performance monitoring frameworks. Some also propose that countries consider codifying PVS-related responsibilities in national legislation or strategic plans, which could help secure long-term political and financial support. Others suggested adding LF as a national notifiable disease to elevate its importance and to impose a legal obligation to take public health action upon receipt of a surveillance signal.

## 4. Discussion

This study provides insights into the practical and systemic challenges of implementing LF PVS in PICTs. By combining structured prioritisation through an NGT with broader perspectives from an expert workshop, we identified not only recurring barriers but also frontline staff suggestions for strategies to improve the feasibility, sustainability, and acceptability of PVS.

A consistent theme across both engagements was that the success or failure of PVS cannot be reduced to a single issue. Instead, implementation reflects the interplay of context-specific, structural, and political influences. This complexity necessitates moving beyond simple linear problem–solution frameworks toward more systems-based approaches and highlights the need for settings-specific design of PVS strategies, a point reflected in the new (2025) second edition of the WHO Monitoring and epidemiological assessment of mass drug administration in the GPELF manual [10]. Developing user-friendly tools and operational guidance to support context-sensitive planning, such as decision trees or costing calculators, could enable program managers to design appropriate PVS strategies.

Both the NGT and workshop underscored the misalignment between global expectations for sustained PVS and the operational realities faced by PICT officers responsible for delivery. Despite strong encouragement, PVS implementation has often been ad hoc, constrained by resource scarcity, geographic dispersion, and the absence of formal mandates or dedicated funding. We heard that PICTs that achieve elimination universally experience a paradox: success is followed not by sustained investment, but by declining political attention. Raising awareness among senior decision-makers of the risks of LF resurgence is therefore critical. Suggested strategies include high-level advocacy that situates PVS within the broader agenda of regional health security, public recognition of national leadership, and research that directly addresses decision-makers’ priorities, including cost and budget-impact analyses that clarify the health system implications of conducting, or not conducting, PVS.

At the global and regional level, aligning LF PVS with frameworks such as the WHO NTD Roadmap [26], WHO Guidelines on Verification of Measles Elimination in the Western Pacific Region [27], and embedding advocacy and regional planning for PVS implementation within the established governance architecture of the Pacific through the Pacific Public Health Surveillance Network [28] have the potential to foster commitment, sustainability, and accountability. Workshop discussions highlighted that the release of the new WHO guidelines [10], although welcomed, will require accompanying context-relevant guidance and technical assistance in order for them to be adopted as intended. Co-development of PICT-specific supporting material and support from advisors who “understand the Pacific context” was called for.

Findings from both engagements emphasised that the absence of national or regional mandates has left PVS vulnerable to donor cycles, individual champions, or ad hoc opportunities. Embedding surveillance within established frameworks, such as the Pacific Healthy Islands Monitoring Framework [29], or re-envisioning the widely recognised success of the PacELF model to suit current epidemiological realities and support needs could create programmatic continuity and attract donor support.

Integration emerged as theme with participants appreciating the cost-efficiencies such approaches may offer. Embedding PVS into established service platforms, such as NCD screening, workplace health checks, or maternal health services, was seen as a pragmatic approach to reduce marginal costs and increase acceptability. Recent examples of integrated PVS discussed during the expert workshop offer models that may be built upon or replicated.

Participants in both the NGT and the expert workshop identified misunderstandings among leaders and communities about the distinction between elimination and eradication, along with survey fatigue, as barriers. Conversely, leveraging trusted networks, such as churches, was suggested as an opportunity to enhance understanding and participation.

Operational bottlenecks, such as lengthy stock procurement processes and shortages, were also noted as barriers to PVS implementation. These challenges may be alleviated by taking a regional approach to PVS implementation that supports scheduled PVS activities and the forecasting of resource needs. Such an approach would likely support national public health programs in better planning for and integrating LF and f activities to enable requirement forecasting and planning, and through pooled procurement mechanisms supported by WHO and partners.

This study is not without limitations. First, NGT and expert workshop participants attended regional meetings and, while senior and experienced, may not represent the full spectrum of opinions. Furthermore, representatives from Palau, one of the eight PICTs that have eliminated LF as a public health problem, were not represented in the study. Second, the senior authors have conducted prior conceptual and applied research on LF PVS in the Pacific, which may have influenced their views. Third, the

## 5. Conclusions

The small population sizes and remoteness of PICTs present both challenges and opportunities for sustaining LF elimination. A coordinated, programmatic approach to LF PVS, supported by national authorities, development partners, and academic institutions, has the potential to make PVS feasible in PICTs. While the findings of this study are grounded in the PICT context, several lessons are likely transferable to other settings, particularly Small Island Developing States that share similar geographic, workforce, and resource constraints. More broadly, this work contributes novel insights from the perspectives of frontline health practitioners working in an understudied region. Their experiences, rooted in the practical realities of delivering LF PVS, underscore the importance of ensuring that global guidance is not only technically sound but also operationally feasible and responsive to end-user needs.

## Figures and Tables

**Table 1:** Summary of recommended actions, responsible actors, and proposed progress indicators to address critical barriers to post-validation surveillance for lymphatic filariasis.

Theme/Barrier	Operational Actions	Responsible Actors	Progress Indicators
1. Leadership awareness and understanding	Advocacy briefings; policy briefs; orientation sessions; risk communication materials	Ministry of Health leaders; LF programme managers; WHO; regional advisers	No. of briefings; leaders reached; knowledge improvement; inclusion of PVS in policies
2. Technical and financial support for PVS	Secure PVS budget lines; develop investment cases; align with funded programmes; apply for donor funds; design a strategic regional approach to LF elimination.	National budget planners; donors; health programme managers; regional advisors	Existence of budget line; funds allocated; number of funding partners; % funding needs met; development of regional strategy for LF-PVS
3. Context-specific guidance and support for PVS implementation	Adapt WHO guidelines to meet contextual needs/realities; develop Pacific-specific SOPs & toolkits; provide decision trees; build advisory network	LF programme staff; technical partners; WHO; regional networks	No. of guidance documents; training sessions; adoption rate; user satisfaction
4. National capacity & workforce development	Provide in-service training; mentorship & exchanges; embed PVS roles within job descriptions; retain skilled staff	Universities and other training institutions; senior staff; regional mentors; partners	No. of trained staff; mentorship exchanges; retention rates; independent PVS operations
5. Diagnostic tools and supplies	Adopt field-ready antigen tests; consider dry-blood spot-based antibody testing; streamline equipment/test procurement processes; designate and build capacity of regional laboratories	Laboratories; procurement units; field teams; WHO; diagnostic suppliers	Availability of kits; stock-out frequency; turnaround time
6. Integration and community engagement	Identify opportunities for integration of PVS with established, routine health programmes; engage the community to build understanding and demand for PVS	Health service providers, technical advisors; academic institutions; community organisations; education sector	No. of integrated PVS surveys; participation rates; change community awareness and demand for PVS
7. Mandates and accountability for PVS	Articulate PVS obligations; include PVS indicators in reports; classify LF as a notifiable disease; monitoring, evaluation and transparent reporting	Legislators; health authorities; reporting units; policymakers	Mandates enacted; reports with indicators; LF listed as notifiable; compliance rate

## Data Availability

Data are available from the corresponding author.

## Supplementary Materials

The following supporting information can be downloaded at: https://www.mdpi.com/article/10.3390/tropicalmed11020062/s1, Table S1: Nominal Group Technique participants’ scoring, consolidated results and top three themes.

## Abbreviations

The following abbreviations are used in this manuscript:

GPELF	Global Programme to Eliminate Lymphatic Filariasis
NGT	Nominal Group Technique
NTD	Neglected-tropical disease
LF	Lymphatic filariasis
MDA	Mass drug administration
NCD	Non-communicable disease
PacELF	Pacific Programme to Eliminate Lymphatic Filariasis
PICTs	Pacific Island Countries and Territories
PPHSN	Pacific Public Health Surveillance Network
PVS	Post-validation surveillance
SPC	[Secretariat of the] Pacific Community
STEPs and STEPwise	“STEPs” and “STEPwise” refers to the step-by-step structure of the WHO NCD risk factor survey methodology rather than being an acronym
WHO	World Health Organization
