# Supplementary material for: Enablers of Post-Validation Surveillance for Lymphatic Filariasis in the Pacific Islands: A Nominal Group Technique and Expert Elicitation
Source: Trop Med Infect Dis. 2026 Feb 23;11(2):62. doi: 10.3390/tropicalmed11020062 (PMC12944890; doi:10.3390/tropicalmed11020062)
Supplement: Supplementary file 1 [file tropicalmed-11-00062-s001.zip › tropicalmed-4112223-supplementary.pdf]

Supplementary Materials

Table S1. Nominal Group Technique participants' scoring, consolidated results and top three themes.

Themes	Votes from each participant (participant number)								Sum of scores	Per cent of total votes	Ranked priority (by sum of scores; top 3)	Sum of participants who allocated any votes	Rank priority (by sum of votes)	Sum of participants who ranked the theme in their top two	Rank priority (by sum of in top 2 themes)
	**1**	**2**	**3**	**4**	**5**	**6**	**7**	**8**							
Top 3															
Limited national health leaders' understanding of and commitment to conduct PVS, or sense of risk	8	5	2	0.5	5	2	3	2	27.5	34%	#1	8	#1	7	#1
Insecurity of resources to conduct PVS	1	3	3	2	0	2	0	3	14	18%	#2	6	#3	6	#2
Access to context-appropriate guidelines for PVS implementation	0	1	1	1.5	2	2	1	2	10.5	13%	#3	7	#2	4	#3
Other															
Limited national capacity for PVS implementation	1	1	1	1.5	0	1	2	0	8.5	11%		6		2	
Overcomplex surveillance/diagnostic tools and processes	0	0	0	0.5	2	1	1	1	5.5	7%		5		1	
Build community demand for PVS	0	0	1	2	0	0	0	1	4	5%		3		1	
Reorient PVS back to a community-led/community-valued public health intervention	0	0	0	1	1	1	1	0	4	5%		4			
Establish requirements/mandates for PVS	0	0	0	0.5	0	1	1	1	3.5	4%		4			
Ad-hoc nature of external technical support	0	0	1	0.5	0	0	1	0	2.5	3%		3			
Poor integration with other surveillance processes	0	0	1	0	0	0	0	0	1	1%		1			
